# Editorial: Perinatal Palliative Care Comes of Age

**DOI:** 10.3389/fped.2021.709383

**Published:** 2021-06-22

**Authors:** Brian S. Carter, Elvira Parravicini, Franca Benini, Paola Lago

**Affiliations:** ^1^Department of Medical Humanities & Bioethics, University of Missouri-Kansas City School of Medicine, Kansas City, MO, United States; ^2^Columbia University Medical Center, New York City, NY, United States; ^3^Pediatric Pain and Palliative Care Service, Department of Women's and Children's Health, University Hospital Padua, Padua, Italy; ^4^Department for Mother and Child Health, Veneto Regional Centre for Pain and Paediatric Palliative Care and the Children's Hospice, Padua, Italy; ^5^Neonatal Intensive Care Unit, Woman's and Child's Department, Ca' Foncello Hospital-Treviso, Treviso, Italy

**Keywords:** palliative care, neonatology, neonatal ICU, end-of-life, moral distress

Advances in medical technology have allowed for the prenatal detection of anatomical congenital anomalies leading to well-recognized patterns of concern that may involve any organ system. They may be due to chromosomal or other genetic differences. Or they may result of altered maternal-placental-fetal metabolism, immune function, or environmental exposure to teratogens. The affected fetus, and newborn, is said to have a life-limiting condition and postnatal life may be brief. Even with resuscitation and admission to a neonatal ICU (NICU), hospitalization may be prolonged only to lead to technology dependence, and adverse prognoses.

Perinatal palliative care (PnPC) has grown out of the field of hospice and palliative medicine (HPM) and increasingly available services of pediatric HPM. The goal of PnPC is to address and support the family living with a concerning, life-limiting, or life-threatening fetal diagnosis. Their pregnancy narrative has been disrupted—their story, in a way, has been broken. PnPC offers a comprehensive interdisciplinary approach for the care of the parents during their continued pregnancy, to help them redirect their attention to deal with the news that they have received and consider the story that they can best live with moving forward for their newborn and themselves. For each family, and unique newborn, care is directed toward achieving a state of comfort by satisfaction of the newborn's basic needs, pain and symptom management, and supportive psychosocial and spiritual care for family members across generations in a culturally sensitive framework. PnPC also provides support for professionals.

While PnPC addresses the perinatal journey that starts prenatally with a concerning fetal diagnosis, it continues at birth and may accommodate resuscitative measures to smooth neonatal transition and optimize time with a family while the baby is alive—even when invasive interventions and a lengthy NICU stay may not be the elected treatment paradigm. PnPC provides accompaniment for the baby and his/her family as an inpatient and may continue into the post-discharge outpatient timeframe. It also may be an option that offers a plan of care directed at improving the baby's quality of life when cure-oriented and life-prolonging treatment is no longer the goal of care for a baby who has been treated in the NICU.

The interdisciplinary approach that PnPC brings is one of collaboration across professional disciplines, including high-risk obstetricians, neonatologists, and other specialists, in accordance with each specific diagnosis. Services to help provide comfort to the baby (lactation consultant, feeding therapists, speech pathologist, OT/PT) and emotional, psychological, and spiritual support to families (Social Work, Psychology, Child Life, and Chaplaincy or engaging the family's faith community) are summoned to help. Yet, while there are over 300 programs of PnPC reported internationally, there is no current standard of PnPC team composition or service provision for the fragile population it addresses.

Here we provide 13 peer-reviewed manuscripts aimed at addressing much of what PnPC is, offers, and considers, its interdisciplinary team nature, and the need for ongoing study and development.

Beginning with a concerning fetal diagnosis, Marc-Aurele describes for us in her article, the Decisions Parents Make When Faced with Potentially Life-Limiting Fetal Diagnoses and the Importance of Perinatal Palliative Care how families consider gaining additional diagnostic information and the value of additional pediatric specialty consultation to inform parents of what to expect as they continue a pregnancy. She introduces birth planning, which is further elaborated upon by Cortezzo et al. who expound on the conversations that precede and follow the development of any birth plan, comparing it to other forms of Advance Directives. In Perinatal Palliative Care Birth Planning as Advance Care Planning Cortezzo relates how birth planning allows families to express their fears, values, hopes, and wishes.

Symptom management follows as Cortezzo and Meyer review of caring for newborns and their end-of-life symptoms in Neonatal End-of-Life Symptom Management. This subject is also addressed in a separate chapter, End-of-Life Care for Neonates: Assessing and Addressing Pain and Distressing Symptoms by Haug et al. Importantly, the paucity of rigorous evidence-based research on therapeutics is noted, and the PnPC clinician may be at his best when he adopts practices from other populations while remaining considerate of the constraints and context of the neonate's physiology. Kain RN addresses the importance of various cultural and spiritual considerations that caregivers should understand in their approach to patients and families of varied faith traditions. Perinatal Palliative Care: Cultural, Spiritual, and Religious Considerations for Parents—What Clinicians Need to Know is a useful summation of many, though understandably not all, such traditions.

An excellent model of caring for fragile newborns with anticipated short lives follows in the perspective piece by Wool and Parravicini. In The Neonatal Comfort Care Program: Origin and Growth Over 10 Years they relate the development of the program at Columbia University in New York and how it has grown to serve countless newborns and families in sharing meaningful, humane, and comfortable time together—even when life for the newborn is brief. Another model of PnPC is reported in a perspective piece by Locatelli et al. from Bologna, Italy. “Percoso Giacomo”: An Italian Service of Perinatal Palliative Care. Named for a palliative pathway taken by the family of little Giacomo, this chapter provides an excellent review of key pieces in making PnPC functional.

For many around the world, it will be important to understand how to develop a perinatal palliative care program. While this is understandably determined in some manner by local practices in obstetrics and neonatal care, as well as resources, a nice review of essential mindsets, materials and models should be welcome by all. Lago et al. present such material in Summary of the Key Concepts on How to Develop a Perinatal Palliative Program. This theme is further given attention with the presentation of a specific model of development by Bolognani et al. from Italy in their brief report, Development of a Perinatal Palliative Care Model at a Level II Perinatal Center Supported by a Pediatric Palliative Care Network.

The potential development of moral distress in professional staff is very real within the neonatal ICU caregivers. Mills and Cortezzo provide a superlative paper that any physician or nurse would benefit from reading entitled, Moral Distress in the Neonatal Intensive Care Unit: What Is It, Why It Happens, and How We Can Address It. Additional address of these and broader measures of caring for the caregiver are found in an excellent scoping review by advance practice nurse Grauerholz et al. Fostering Vicarious Resilience for Perinatal Palliative Care Professionals.

No address of PnPC would be complete without addressing barriers to palliative care. Benini et al. provide key insights to such barriers in the perinatal context in their opinion paper, Barriers to Perinatal Palliative Care.

As PnPC programs develop, work through, and overcome barriers, and experience success, it is important to be able to evaluate their impact and, in many ways, “prove” their value to healthcare organizations, hospitals, and health care professionals. Jennifer et al. present an evaluation of the impact of a 3-day training course for interdisciplinary clinicians. Assessment of Healthcare Professionals' Self-Perceived Competence in Perinatal/Neonatal Palliative Care After a 3-Day Training Course. It is important that any future training events be similarly evaluated to assess participant confidence, competence, and best practices.

We sincerely hope that this collection of works from around the world will help give confidence to those needing it, direction for those on the journey of developing PnPC programs and understanding to all.

## Author Contributions

BC, EP, FB, and PL conceived the paper. BC created the first draft. All authors contributed to edits and accepted the final copy of the manuscript.

## Conflict of Interest

The authors declare that the research was conducted in the absence of any commercial or financial relationships that could be construed as a potential conflict of interest.

